# Experience Reduces Surgical and Hardware-Related Complications of Deep Brain Stimulation Surgery: A Single-Center Study of 181 Patients Operated in Six Years

**DOI:** 10.1155/2018/3056018

**Published:** 2018-07-22

**Authors:** Mehmet Sorar, Sahin Hanalioglu, Bilge Kocer, Muhammed Taha Eser, Selim Selcuk Comoglu, Hayri Kertmen

**Affiliations:** ^1^Department of Neurosurgery, Diskapi Yildirim Beyazit Training and Research Hospital, Health Sciences University, Ankara, Turkey; ^2^Department of Neurology, Diskapi Yildirim Beyazit Training and Research Hospital, Health Sciences University, Ankara, Turkey

## Abstract

**Objective:**

Deep brain stimulation (DBS) surgery has increasingly been performed for the treatment of movement disorders and is associated with a wide array of complications. We aimed to present our experience and discuss strategies to minimize adverse events in light of this contemporary series and others in the literature.

**Methods:**

A retrospective chart review was conducted to collect data on age, sex, indication, operation date, surgical technique, and perioperative and late complications.

**Results:**

A total of 181 patients (113 males, 68 females) underwent DBS implantation surgery (359 leads) in the past six years. Indications and targets were as follows: Parkinson's disease (STN) (*n*=159), dystonia (GPi) (*n*=13), and essential tremor (Vim) (*n*=9). Mean age was 55.2 ± 11.7 (range 9–74) years. Mean follow-up duration was 3.4 ± 1.6 years. No mortality or permanent morbidity was observed. Major perioperative complications were confusion (6.6%), intracerebral hemorrhage (2.2%), stroke (1.1%), and seizures (1.1%). Long-term adverse events included wound (7.2%), mostly infection, and hardware-related (5.5%) complications. Among several factors, only surgical experience was found to be related with overall complication rates (early period: 31% versus late period: 10%; *p*=0.001).

**Conclusion:**

The rates of both early and late complications of DBS surgery are acceptably low and decrease significantly with cumulative experience.

## 1. Introduction

Since its introduction in 1987, deep brain stimulation (DBS) has become an effective treatment modality in the management of movement disorders [1, 2]. Clinical trials have proven its efficacy in Parkinson's disease (PD) and other hyperkinetic diseases [2–4]. In recent years, its indications have been broadened by successful applications in many other neuropsychiatric disorders [5].

As in all surgical operations, this procedure is not without complications and problems. In addition to operative complications, inherent to the technique are hardware- and stimulation-related adverse events, which have increasingly been recognized in the literature as the experience has exponentially grown in the past years [6–10]. Here, we present our experience with 181 patients undergoing DBS placement and discuss strategies to minimize complications and adverse events in the light of this contemporary series and others in the literature.

## 2. Materials and Methods

### 2.1. Patients

All patients who had undergone DBS implantation surgery at Diskapi Yildirim Beyazit Training and Research Hospital between 2012 and 2017 were included in this retrospective study. Primary diagnoses and surgical targets were as follows: Parkinson's disease (subthalamic nucleus (STN)), dystonia (globus pallidus interna (GPi)), and essential tremor (ET) (ventral intermediate nucleus of the thalamus (Vim)). All procedures were performed by the same surgical team (MS and HK). Patient data were extracted from electronic health records, patient charts, radiological images, and manufacturers' records of hardware implantations. The DBS devices were primarily St. Jude Medical neuromodulation system (*n*=159, all PD patients); however, Medtronic implants were also used in some patients (*n*=22, all dystonia and ET patients). All patients were referred by neurologists at the Movement Disorders Clinic, and the DBS treatment decision was made jointly by the multidisciplinary team.

Patient data were analyzed retrospectively for the demographics, operative details, and the occurrence of early postoperative and long-term adverse events. Early perioperative period was defined as the first 30 days after implantation surgery, whereas the long-term as the period beyond the first 30 postoperative days. Surgery-related complications were defined as complications that occurred during or within 30 days of surgery and directly related to the operative procedure itself (hemorrhages, seizures, etc.). Hardware-related complications were defined as adverse events due to the problems in hardware components or body parts (e.g., skin) in direct contact with them, irrespective of the time of occurrence. Complications occurring after the surgery for internal pulse generator (IPG) replacement due to depletion were excluded.

### 2.2. Surgical Procedure

Preoperative volumetric MR imaging is done one day prior to surgery. On the day of surgery, a stereotactic frame (ZD stereotactic system) is placed, and a patient undergoes volumetric CT imaging which is fused by preoperative volumetric MR images using Brainlab planning software (BrainLab AG). Indirect targeting is completed based on reference to the anterior commissure-posterior commissure line. The trajectory is determined to avoid cortical vessels (using TOF MRA) and, if possible, the lateral ventricle, usually choosing burr hole locations 4-5 cm lateral from the midline at the coronal suture. The surgery is performed under local anesthesia (except for dystonia). Microelectrode recording (MER) was routinely performed for all targets (the number of inserted electrodes ranging 1 to 3). Intraoperative test stimulation was performed to verify the target accuracy and the lack of sustained side effects. The placement of lead extensions and IPG was performed during the same time as lead implantation. Postoperative MRI was routinely performed to verify the location of leads. In addition, cases suspected for intracranial hemorrhage underwent head CT.

### 2.3. Statistical Analysis

All statistical analyses were performed using the standard statistical software (SPSS Statistics, version 22.0; SPSS Inc.). Risk factors for the occurrence of adverse effects such as patient's age, diagnosis, and date of surgery (early period: 2011–2014; late period: 2014–2017) were analyzed with logistic regression. Student's *t*-test with equal variances was used to compare age at implantation between patients with adverse effects and those without. Evaluation of differences between patients with adverse events depending on surgical experience was performed using the Fisher exact test. A *p* value below 0.05 was considered as statistically significant.

## 3. Results

### 3.1. Demographic Data

One hundred eighty-one patients received 359 new DBS leads in a total of 181 stereotactic procedures. Two hundred and one IPG replacement procedures were performed during the study period. Of the newly implanted leads and IPGs, a majority (85%) were manufactured by St. Jude Medical and others by Medtronic.

One hundred fifty-nine patients suffered from PD, 13 from dystonia, and 9 from essential tremor. Targets were bilateral STN in all PD, bilateral GPi in all dystonia, and bilateral Vim in all ET patients except for three cases in whom unilateral DBS implantation was done. Patients' age ranged from 9 to 74 years (mean: 55.2 ± 11.7 years), and 62.4% were male. A vast majority of the patients (87.8%) had follow-up of more than 1 year (mean: 3.4 ± 1.6 years). No patients have been lost to follow-up.

### 3.2. Perioperative Events

A summary of perioperative and long-term complications is presented in Table 1. No mortality or permanent morbidity was observed after surgery in this series. Perioperative complications were detected in a total of 17 patients (9.4%). The most common perioperative adverse event was postoperative confusion/delirium, which was seen in 12 patients (6.6%). All these patients had PD and underwent bilateral STN DBS implantation. Confusion resolved in all patients with medication during hospitalization approximately within a week. Two patients (1.1%) had seizures in the early postoperative period and treated with a single antiepileptic drug (AED). They were seizure-free at the 6th month after surgery, and AED was discontinued.

**Figure 1:**
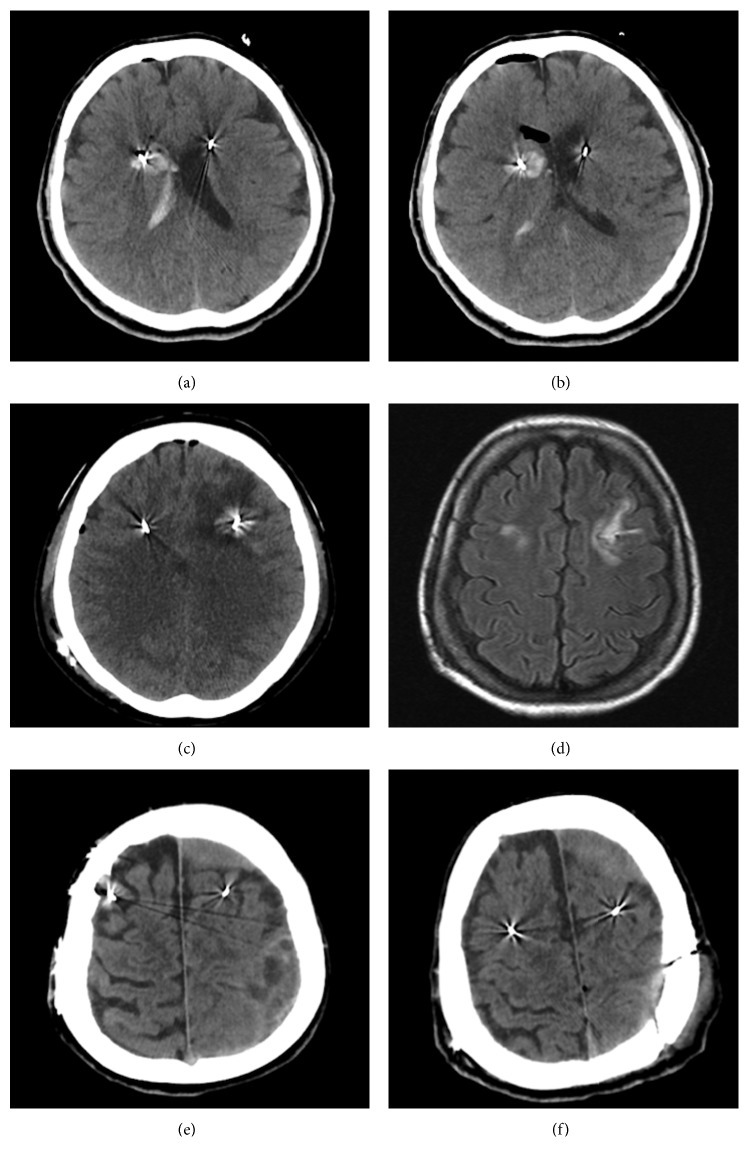
Operative complications due to deep brain stimulation surgery. (a, b) Right caudate hematoma with intraventricular hemorrhage. (c) Venous hemorrhagic infarction around the left-sided DBS lead; (d) 6-month follow-up FLAIR MRI. (e, f) Pre- and postoperative images of the left-sided chronic subdural hematoma over the parietal cortex. Note that the frontal component of the cSDH was left intact to avoid iatrogenic injury to the lead.

The most common severe complication of surgery was hemorrhage (*n*=5, 2.8%), including four cases of intracerebral hematoma (ICH) (2.2%) and one case of venous hemorrhagic stroke (0.6%). In two of them (1.1%), small hematomas along the leads were detected with no or only slight transient symptoms. However, one patient developed moderate paresis in both upper extremities (with a few hours interval) due to slowly evolving hemorrhages, possibly of venous origin, around both leads. The patient was treated conservatively and recovered completely in 6 months. In another patient, an ICH was suspected intraoperatively due to development of left hemiparesis following lead implantation. Immediate postoperative CT scan showed right caudate hematoma with associated intraventricular hemorrhage (Figures 1(a) and 1(b)). Then, the IPG placement was postponed, and the patient was transferred to the ICU where he was treated conservatively. One week later, his symptoms and hematoma largely resolved and he underwent IPG placement with no further problems.

Another patient developed slight right hemiparesis and dysphasia at postoperative day 3. She underwent CT scan which showed a left frontal venous hemorrhagic stroke (Figures 1(c) and 1(d)) evidenced by a relatively large infarct area around a small hemorrhagic component surrounding the lead. This patient recovered completely with no sequelae within 3 months.

One patient with essential tremor had ischemic stroke (0.6%). He became dysphasic immediately after the DBS implantation. Diffusion-weighted MRI showed acute diffusion restriction in the left frontal cortex around the electrode. He was also treated conservatively and recovered completely within a month.

### 3.3. Long-Term Events

Those events were described as adverse events that occurred later than 30 days after surgery. Wound and hardware complications constituted a majority of those problems.

#### 3.3.1. Wound Complications

Infection was the most common wound complication (*n*=11, 6.1%) followed by allergic inflammation (*n*=2, 1.1%). A majority of those cases were self-limiting and managed with antibiotics. Four patients (2.2%) underwent surgery for debridement and repair of wound erosion and/or dehiscence without need for system removal. Scalp reconstruction was performed in two of them. Five patients (2.8%) required removal of the IPG due to infection or recurrent allergic inflammation at the IPG site. Two of them had infections due to trauma; two had abscess formation, and another had sterile seroma around the IPG. Revision surgery was performed depending on the system components affected (either IPG only or IPG + lead extensions).

#### 3.3.2. Hardware Complications

Lead position was found to be suboptimal (>2 mm change from the initial position) in only two patients (1.1%) as confirmed by the comparison of late CT with early postoperative MRI and CT (fused images were used to identify the exact anatomic location of the leads). However, fracture or disconnection of leads or lead extensions (including component malfunction as indicated by high impedance in the system) was relatively common in this series (*n*=8, 4.4%). In most cases (*n*=7), the problem was detected in the lead extensions and only they were replaced. Twiddler's syndrome was diagnosed in one of them. In one patient, iatrogenic injury to the DBS system (during an operation for a trauma in another center) resulted in the revision of the entire system including leads and extensions and IPG revision.

#### 3.3.3. Other Complications and Problems

Two patients suffered from chronic subdural hematomas (cSDHs). One patient presented with speech disturbance and slight right-sided weakness 6 weeks after the DBS implantation. A CT scan showed a cSDH with multiple septations over the left hemisphere (Figure 1(e)). He underwent craniotomy to evacuate hematoma with preservation of DBS hardware (Figure 1(f)). He had an uneventful postoperative course and complete recovery. Another patient had a bilateral cSDH (more on the left than the right) following a mild head trauma 3 years after initial DBS surgery. He underwent an endoscope-assisted evacuation of the cSDH through burr holes, with preservation of DBS hardware. This patient also recovered completely.

### 3.4. Risk Factors

**Figure 2:**
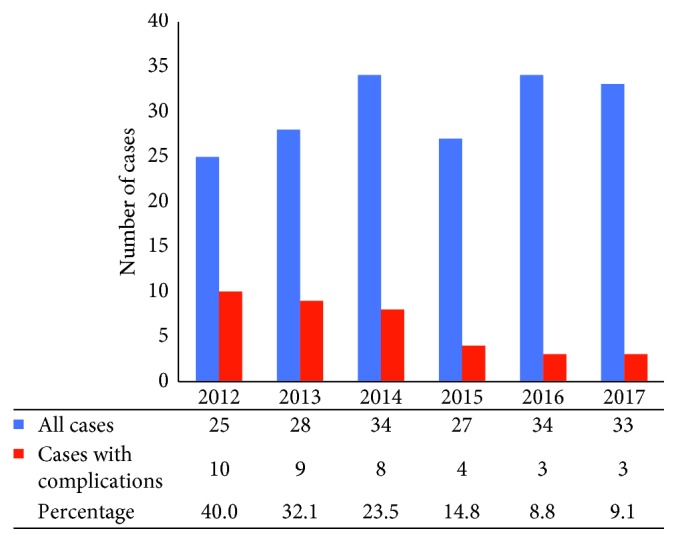
Cumulative experience reduces the rate of complications following DBS surgery. The graph shows relatively constant annual caseload but remarkable reduction in the percentage of cases with complications throughout the years.

Potential predictors of adverse events were analyzed. Neither age nor sex was found to be related to risk of complications. Furthermore, there was not significant difference between age of the patients with and without complications (54.9 ± 9.9 versus 56.7 ± 9.3, *p*=0.319). The primary diagnosis/indication, anatomical target, and device manufacturer did not appear to affect complication rates (20.1% versus 22.7%, *p*=0.776), except for confusion occurring only in PD patients who underwent bilateral STN DBS. On the contrary, surgical experience seems to be directly related to overall complication rates (early period (2012–2014): 31.0% versus late period (2015–2017): 10.6%, *p*=0.001). A drastic reduction in complication rates is observed throughout the years (Figure 2).

## 4. Discussion

Deep brain stimulation surgery has been utilized for the treatment of medically intractable movement disorders for more than two decades. Various clinical series have shown that it is a safe procedure with low rates of complications and adverse events, particularly in the experienced large-volume centers [8–10]. Here, we investigated occurrences and risk factors of surgical and hardware-related complications of DBS surgery performed by a single primary surgeon in a single center.

Early adverse events within 30 days of surgery are generally attributed to surgery [11, 12]. Literature regarding surgical complications in DBS surgery is extensive [6–16]. Incidences of overall short-term or surgery-related complications vary considerably between 2 and 20% depending largely on the definition of adverse events [12–14]. In our series, 11% of the patients experienced early complications. The most common surgical adverse event was perioperative confusion (6.6%). Various studies have reported similar rates (3–7%) regarding postoperative mental status change, confusion, or delirium [9, 13, 15, 16]. It usually has a favorable course and resolves spontaneously or with adequate medication within a few days of hospitalization.

Seizures are also important complications of DBS surgery [17]. We had two patients (1.1%) having seizures following the hardware implantation. Their seizures were controlled with a single antiepileptic drug, which were discontinued 6 months after the operation. Some groups have reported higher frequencies (4–7%) of postoperative seizures, nevertheless, in virtually all series; their outcomes were quite favorable posing no serious risk for DBS hardware or chronic epilepsy [17–19].

Although it is not very common, hemorrhagic events are the most feared ones of operative complications [20, 21]. Various large series reported incidences of hemorrhagic complications, mainly in the form of intracerebral hemorrhage, between 0.5 and 5% [8, 20–24]. Immediate postoperative imaging could miss an ICH as some ICHs develop or enlarge in delayed fashion [24]. Furthermore, more than half of ICHs are asymptomatic and usually require no intervention at all [8]. However, when large and symptomatic, ICH is one of the most dreadful complications of DBS surgery. Fortunately, such a symptomatic ICH is rarely encountered (<1-2%) and usually has a relatively benign course [6, 8, 25]. In our series, we observed two such cases (1.1%), both of whom recovered fully with medical treatment and observation only. On the contrary, we had to operate on a patient who presented with chronic subdural hematoma 1.5 months after the operation. Extensive membranes within the cSDH prevented us from doing minimally invasive drainage via burr holes [26]; therefore, we had to do craniotomy to evacuate hematoma while preserving the DBS system.

Stroke, both ischemic and hemorrhagic [27], is also a rare but serious complication after DBS implantation. We encountered one ischemic and one venous hemorrhagic infarct during the postoperative course. Both patients have eventually recovered with supportive medical treatment without necessitating hardware removal. Careful planning on preoperative images is a prerequisite for avoidance of vascular injury [23]. We routinely use TOF MRI in image fusion to track vascular structures, while other groups reported successful use of CT angiography [28], contrast-enhanced MRI [25], and SWI-MR venography [29] separately or in combination for this purpose. Whether microelectrode recording increases the risk of vascular injury is also a matter of debate [22, 23], with some groups reporting a significant drop in the rates of hemorrhagic complications after minimizing or refraining from microelectrode recording [21, 30]. Likewise, we usually perform MER with only two electrodes (between one and three), which could possibly reduce the risk of vascular injury in comparison to multiple microelectrode insertion techniques.

Late complications might be equally, or even more, problematic [31]. Wound complications, particularly infection, comprise the majority of these complications [32, 33]. A recent systematic review of hardware-related complications of DBS surgery revealed that infections (5.12%) were followed by lead migration (1.60%), fracture or failure of the lead or other parts of the implant (1.46% and 0.73%, resp.), IPG malfunctions (1.06%), and skin erosions without infections (0.48%). The authors also indicated that new indications for DBS, including Tourette's syndrome, cluster headache, and refractory partial epilepsy, were found to pose a higher risk of hardware-related infections than established indications such as Parkinson's disease [33]. In our series, slightly higher proportion (10%) of the patients suffered from the wound problems. But fortunately, most of the infections respond to antibiotherapy and/or surgical debridement. Only a fraction of these patients had intractable infection or inflammation that made removal of hardware components (mostly extension and IPG) inevitable. With the advent of new technologies, better surgical techniques, and prevention strategies [10, 34, 35], the rates of such serious wound complications have diminished considerably. Also, alternative medical and surgical strategies have been used to tackle these complications once occurred [36, 37].

Cumulative probability of hardware-related complications increases with longer follow-up durations [6, 38, 39]. In our series, we observed only two instances of lead malposition (1.1%), which represent a lower rate than that in most published series [7, 8, 16, 38, 40]. Of note, we confirm position of leads with routine immediate postoperative MRI and can check for lead migration using image fusion with CT scans during follow-up. On the contrary, fracture or disconnection of lead extensions, but not leads themselves, was more common in our series, possibly due to frequent traumatic incidents that those patients experienced. Replacement of lead extensions and IPG yielded good results in these patients.

Regarding the risk factors for surgical and hardware-related complications, many factors have been determined in previous studies such as advanced age, male sex, primary diagnosis, hypertension, obesity, and anemia [7, 12, 22]. In this series, we did not observe any effect of age, sex, or primary diagnosis on complication rates in line with findings of some other studies [14, 41]. In fact, the only factor we could determine to relate to complications was surgical experience [42, 43]. The fact that both multidisciplinary team dealing with and the protocols used for planning and execution of DBS implantation in our institute have remained unchanged for the past six years makes this series a unique one to demonstrate the impact of accumulating surgical experience in reducing complication rates over the years.

As the indications and availability of DBS expand, the number of patients suffering from its complications will also increase [33]. Therefore, our main goal should be to minimize the complication rates with the help of better technologies and more efficient surgical techniques and, most importantly, to better understand the mechanisms and cumulative experience in the field [43, 44]. This study underscores the critical role of surgical experience in reducing complications and thus shows that instead of making DBS surgery widely available, centralization at several large-volume centers could yield better results with fewer complications.

## 5. Conclusions

A wide range of complications and adverse events are associated with deep brain stimulation surgery. Neurosurgeons should inform patients about these potential problems and be prepared for them before, during, and after the surgery. Advances in our understanding, related technology, and surgical techniques have led to a dramatic decrease in the rates of these adverse events in the past two decades. Our study affirms the role of cumulative experience at individual centers in reducing the rate of complications. Further studies with larger cohorts are warranted to establish other risk factors and develop better strategies to tackle problems related to DBS surgery.

## Figures and Tables

**Table 1:** Summary of the early postoperative and long-term complications in the current series.

Complication/adverse event	Number	%
*Early perioperative complications (<30 days)*		
Confusion/alteration in mental status	12	6.6
Hemorrhages	5	2.8
Intracerebral hematoma	4	2.2
Asymptomatic	2	1.1
Symptomatic	2	1.1
Venous hemorrhagic infarct	1	0.6
Ischemic infarct	1	0.6
Seizure	2	1.1

*Long-term complications (>30 days)*		
Wound complications	13	7.2
Infection/dehiscence	11	6.1
Inflammation/allergy	2	1.1
Hardware-related complications	10	5.5
Lead malposition/migration	2	1.1
Fracture/disconnection (lead or lead extension)	8	4.4
Other complications	2	1.1
Chronic subdural hematoma	2	1.1

## Data Availability

The data used to support the findings of this study are available from the corresponding author upon request.
